# *Hdac7* promotes lung tumorigenesis by inhibiting Stat3 activation

**DOI:** 10.1186/s12943-017-0736-2

**Published:** 2017-11-10

**Authors:** Yubin Lei, Lingling Liu, Shujing Zhang, Shicheng Guo, Xiaoqing Li, Jiucun Wang, Bo Su, Yuchao Fang, Xiaofeng Chen, Hengning Ke, Wufan Tao

**Affiliations:** 1 Obstetrics & Gynecology Hospital and State Key Laboratory of Genetic Engineering and Institute of Developmental Biology and Molecular Medicine, Collaborative Innovation Center of Genetics and Development, School of Life Sciences, Fudan University, Shanghai, China; 2 MOE Key Laboratory of Contemporary Anthropology and Collaborative Innovation Center of Genetics and Development, School of Life Sciences, Fudan University, Shanghai, China; 3 Shanghai Pulmonary Hospital, Tongji University, Shanghai, China; 4 Huashan Hospital, Fudan University, Shanghai, China; 5 Cancer Research Institute, General Hospital, Ningxia Medical University, Yinchuan, China

**Keywords:** *Hdac7*, *Stat3*, Acetylation & phosphorylation, Lung cancer

## Abstract

**Background:**

Lung cancer is the leading cause of cancer death worldwide. However, the molecular mechanisms underlying lung cancer development have not been fully understood. The functions of histone deacetylases (HDACs), a class of total eighteen proteins (HDAC1–11 and SIRT1–7 in mammals) that deacetylate histones and non-histone proteins, in cancers are largely unknown.

**Methods:**

*Hdac7* ^+/−^/*K-Ras* mice and *HDAC7*-depleted human lung cancer cell lines were used as models for studying the function of *Hdac7* gene in lung cancer. Kaplan-Meier survival analysis was performed to explore the relationship between *HDAC7* expression and prognosis of human lung cancers. Recombinant lentivirus-mediated in vivo gene expression or knockdown, Western blotting, and pull-down assay were applied to investigate the underlying molecular mechanism by which *Hdac7* promotes lung tumorigenesis.

**Results:**

The number and burden of lung tumor were dramatically reduced in *Hdac7* ^+/−^/*K-Ras* mice compared to control *K-Ras* mice. Also, in *Hdac7* ^+/−^/*K-Ras* mice, cell proliferation was significantly inhibited and apoptosis in lung tumors was greatly enhanced. Similarly, cell proliferation and anchorage-independent growth of human lung cancer cell lines expressing shHDAC7 were also significantly suppressed and apoptosis was dramatically elevated respectively. Mechanistic study revealed that *Hdac7* mutation in mouse lung tumors or *HDAC7* depletion in human tumor cell lines resulted in significantly enhanced acetylation and tyrosine-phosphorylation of Stat3 and HDAC7 protein directly interacted with and deacetylateed STAT3. The *Hdac7* mutant-mediated inhibitory effects on lung tumorigenesis in mice and cell proliferation/soft agar colony formation of human lung cancer cell lines were respectively reversed by expressing *dnStat3*. Finally, the high *HDAC7* mRNA level was found to be correlated with poor prognosis of human lung cancer patients.

**Conclusion:**

Our study suggests that *Hdac7* promotes lung tumorigenesis by inhibiting Stat3 activation via deacetylating Stat3 and may shed a light on the design of new therapeutic strategies for human lung cancer.

**Electronic supplementary material:**

The online version of this article (10.1186/s12943-017-0736-2) contains supplementary material, which is available to authorized users.

## Background

Histone deacetylases (HDACs) are a class of total eighteen proteins (HDAC1–11 and SIRT1–7 in mammals) that deacetylate histones and non-histone proteins [1]. HDACs play very important roles in diverse biological processes and related diseases such as cancers. High levels of HDACs is frequently associated with advanced cancers and poor prognosis [2]. However, HDACs also display tumor suppressive effects in some cancers, e.g. an *HDAC2* truncating mutation was observed in human epithelial cancers [3]. Low expression of HDAC10 is associated with poor prognosis of lung and gastric cancers [4, 5]. *Hdac2* ^−/−^ mice display a decreased intestinal tumors [6] while liver-specific *Hdac3* KO mice develop hepatoma [7]. Furthermore, *Hdac1* and *Hdac2* function as tumor suppressors on preleukemic stage, but oncogenes for leukemia maintenance in PML-RAR-mediated mouse acute promyelocytic leukemia [8].

HDAC7 is a member of the HDAC family. Studies conducted on cell culture level by silencing or overexpressing *HDAC7* have shown that *HDAC7* is involved in the regulation of cell proliferation, apoptosis, differentiation and migration. *Hdac7* ^−/−^ mice are embryonic lethal due to a failure of angiogenesis (rupture of blood vessels) [9]. There are only a few reports studying the role of *HDAC7* in cancers and even these scarce results were controversial. High HDAC7 protein level was observed in 9 out of 11 human pancreatic cancers [10]. *HDAC7* together with *HDAC1* is specifically over expressed in breast cancer stem cells (CSCs) and necessary to maintain CSCs [11]. High *HDAC7* expression was associated with poor prognosis of 74 children with B-lineage CD10-positive acute lymphoblastic leukemia (ALL) (≥95% common-ALL/pre-B ALL) [12]. All these results suggest an oncogenic function of *HDAC7* in these human cancers. However, low *HDAC7* expression was also observed in 75% of 28 pro-B-ALL samples [13] and reported to be associated poor prognosis of lung cancer patients [4]. This suggests a potential tumor suppressive function of *HDAC7* in some other cancers and/or at different stages of cancer development.

Signal transducer and activator of transcription 3 (STAT3) is a member of the STAT protein family. STAT3 activity is modulated by both acetylation and phosphorylation. In response to cytokines or growth factors, STAT3 is tyrosine-phosphorylated by receptor or nonreceptor kinases, forms dimmer and translocates into nucleus to activate the transcription of target genes implicated in a broad range of biological processes. In response to extracellular environmental factors including cytokines and nutrition, STAT3 acetylation is modulated by histone acetyl transferases/HDACs, e.g. p300/HDAC3, and therefore affecting its dimerization, phosphorylation, DNA binding and transactivation [14]. The STAT3 mutated at key acetylation sites abolishes its ability of dimerization, and consequently impairs its tyrosine-phosphorylation and so forth [14, 15]. STAT3 is thought to potently promote oncogenesis in a variety of tissues. The persistent tyrosine phosphorylation of STAT3 (pY-STAT3) and higher expression of STAT3 are observed in many human cancers and are often correlated with an unfavorable prognosis in these patients [16]. However, a growing number of reports also suggest a tumor-suppressive function of STAT3 in some cancers. The pY-STAT3 level is negatively correlated with tumor size and distant metastases of papillary thyroid carcinomas [17, 18]. High pY-STAT3 level is highly correlated with a better prognosis for soft tissue leiomyosarcoma, advanced rectal cancer and nasopharyngeal carcinoma [19–21]. Studies on mouse cancer models also reveal that *Stat3* is a negative regulator for tumor progression in *Apc* mutant mice and in *K-Ras* mice [22, 23]. Furthermore, STAT3 promotes oncoprotein EGFRvIII-induced glial transformation when it forms a complex with *EGFRvIII* and conversely inhibits malignant transformation of astrocytes under *Pten* deficiency condition [24].

The role of *STAT3* in lung cancer development appears complex. The expression of constitutive STAT3 (STAT3C) induced inflammation and adenocarcinomas in mouse lung [25]. STAT3 activation or high expression was initially reported to be associated with poor prognosis of lung cancer patients [26, 27]. However, it has recently been demonstrated that low *STAT3* expression correlated with poor survival and advanced malignancy in human lung cancer patients with smoking history, and disruption of Stat3 signaling enhanced lung tumor initiation and malignant progression in mice [23]. Furthermore, almost at the same time, Zhou, et al., have shown that *Stat3* can function as a tumor suppressor to prevent lung tumor initiation at an early stage of lung tumor development and an oncogene to facilitate lung cancer progression by promoting cancer cell growth at a late stage of lung cancer in the same *K-Ras* mice [28].

Here we report that *Hdac7* functions as an oncogene in lung cancer. Higher HDAC7 protein level is observed in ~44% of human lung cancer samples and higher HDAC7 mRNA level is associated with poor prognosis of lung cancer patients. We also found that lung tumorigenesis was significantly inhibited in *Hdac7* ^+/−^/*K-Ras* mice. *Hdac7* deficiency significantly inhibited proliferation and enhances apoptosis, respectively. The acetylation and phosphorylation of Stat3 were significantly enhanced in tumors from *Hdac7* ^+/−^/*K-Ras* mice and *HDAC7*-depleted human tumor cell lines. We demonstrated that the *Hdac7* mutant-mediated tumor suppression was rescued by expressing *dnStat3* in mouse lung tumors. Finally, our studies showed that HDAC7 directly interacted with and deacetylated STAT3. Our studies may shed a light on the design of new therapeutic strategies for human lung cancer.

## Methods

### Mice

*Hdac7* PB (*PiggyBac*) heterozygote mutant mice (thereafter called *Hdac7* ^+/−^ mice or *Hdac7* mutant mice), carrying an *Hdac7* mutant allele disrupted by insertion of a PB transposon in the intron between exon 1 and 2, was generated on the FVB/NJ background. Mapping information of PB insertion in *Hdac7* gene can be found in the PB mice database (http://idm.fudan.edu.cn/PBmice). *LSL-K-Ras* ^*G12D*^ mice (Stock No. 008179) were previously described [29] and LSL-*K-Ras* ^*G12D*^ allele was introduced into FVB genetic background through breeding with FVB mice for more than six generations (therefore called *K-Ras* mice or control mice). *Hdac7* ^+/−^ mice were crossed with *K-Ras* mice to generate *Hdac7* ^+/−^/*K-Ras* mice. All mice were maintained on 12/12-h light/dark cycles. Experiments were conducted with consent from the Animal Care and Use Committee of the Institute of Developmental Biology and Molecular Medicine at Fudan University, Shanghai, China.

### Lung tumor induction, enumeration and tumor burden analysis

Lung tumors were induced by the method described previously [30]. Briefly, 6-weeks-old *Hdac7* ^+/−^/*K-Ras* and control mice were infected with 5 × 10^7^ plaque-forming units (PFU) of adenovirus (Ad-*Cre*) expressing *Cre* by intranasal inhalation, or 10^6^ transforming unit (TU) of lentivirus (lenti-*Cre* or lenti-*Cre-*2A*-dnStat3*) by tracheal instillation. Six weeks later, mouse lungs were retrieved and tumors on mouse lung surface were counted under dissection microscope. For tumor burden analysis, lungs were perfused through the trachea with 4% paraformaldehyde and fixed overnight followed by standard procedures for paraffin sections and H&E staining. Twelve randomly selected, lung sections/mice were scanned for 6 mice each genotype, total lung area occupied by tumor was measured and tumor burden was calculated as (area of lung section occupied by tumor)/(total area of section) in μm^2^ using Image J (NIH, Bethesda, MD, USA).

### TUNEL assay and EdU incorporation assay

TUNEL assay was performed following the manufacturer’s instructions for a kit (#G3250, Promega, Madison, WI, USA). For EdU (5-ethynyl-2′-deoxyuridine) assay, mice were injected intraperitoneally with 50 mg/kg EdU 6 weeks after Ad-*Cre* infection. Lung tissues were subjected to frozen section followed by EdU staining with a kit (#C10310–2, RiboBio, Guangzhou, China) 24 h after injection.

### Plasmids

pFUW-*Cre*: A *Cre* fragment from MIGR1-*Cre* was cloned into of pFUGW to replace EGFP fragment. Plasmid pFUW-*Cre*-2A-*dnStat3*: A Cre fragment from pCS4-Cre and P2A–*dnStat3* fragment from pCS4-*dnStat3* were cloned into pFUGW to replace EGFP fragment. Plasmid pCMV6-*Stat3*-Flag: a PCR fragment of *Stat3* was cloned into pCMV-Entry and in-frame fused to Flag tag. Plasmid pcDNA3.1-GST-*Hdac7* (aa445–938): PCR fragments of GST and *Hdac7* (aa 445–938) or *Hdac7*AWA (aa 445–938) were cloned into pcDNA3.1 and in-frame fused to Myc tag. Plasmid pLKO.1-shHDAC7: The selected ShHDAC7 targeting sequences were synthesized and cloned into pLKO.1. They are: shHDAC7 #1: ATCCGGGTGCACAGTAAATA, shHDAC7 #2: AAGTAGTTGGAACCAGAGAA, shHDAC7 #3: TCACTGACCTCGCCTTCAAAG.

### Cell culture, virus preparation and infection

293T cells were cultured in as described [31]. Human lung cancer cells A549, H1299, H2009 and H522 were cultured in RPMI 1640 with 10% FBS. Ad-*Cre* was prepared and titrated as previously described [32]. Recombinant lentiviruses were generated by co-transfecting lentivirus expression vectors and package plasmids (pCMV-VSV-G, pRSV-Rev and pMDLg/pRRE) into 293T cells and harvested 48 h later. Harvested lentiviruses for knockdown experiments were stored at −80 °C or directly used to infect human lung cancer cell lines. *Hdac7-*silencing or scrambled cells were selected with puromycin. Lentiviruses for infecting mice were further concentrated by PEG 6000 as described previously [33]. TU of lenti-*Cre* and lenti-*Cre-*2A*-dnStat3* was determine by infecting 293T cells carrying LSL-EGFP transgenes followed the methods published [33].

### MTT assay

Cells were plated at a density of 5000 cells/well in 96-well plate and cultured for desired time periods. The medium was then replaced by 200 μl of fresh RPMI 1640 and 20 μl MTT (5.0 mg/ml) and incubated for 4 h at 37 °C. 100 μl/well DMSO was added before reading OD 570 on a plate reader (Bio-Rad, Hercules, CA, USA).

### Annexin V assay

H1299 cells infected shHAC7 or scrambled lentivirus were gently dissociated with trypsin/EDTA and stained for annexin V and 7AAD using the eBioscience™ Annexin V Apoptosis Detection Kit APC (#88–8007-74, Thermo Fisher Scientific) according to manufacturer’s instructions. The stained cells were immediately analyzed by flow cytometry (FACSCalibur, BD Biosciences). All annexin V positive cells were counted as apoptotic cells.

### Soft agar assay

Soft agar assay was performed as previously described [34]. Briefly, cells were plated at a density of 500 cells/well in 6-well plates and cultured for 14–21 days. Colonies were stained with MTT and counted using Colony Counter software (Tanon, Shanghai, China).

### Quantitative PCR

Total RNA was extracted from wild type (wt) and *Hdac7* mutant mouse lung tissue with TRIzol (Invitrogen, Carlsbad, CA, USA). Quantitative RT-PCR was performed with TaKaRa RNA PCR kit (TaKaRa, Dalian, China) and with Fast SYBR Green QPCR Master Mix on Mx3000P (Stratagene, San Diego, CA, USA) according to the manufacturer’s instructions and data were analyzed with the MxPro software. Expression of *Gapdh* was used as an internal control. The primer sequence is: *Hdac7-*F: CCCAGTGTGCTCTACATTTCCC, *Hdac7-*R: CACGTTGACATTGAAGCCCTC.

### Antibodies, immunoblotting and immunoprecipitation

Antibodies against the following proteins were used for our studies: HDAC7 (#ab53101) and AKAP12 (#ab49849) from Abcam (Cambridge, UK); acetylated lysine (#9441), STAT3 (#8768), Phospho-STAT3 (Tyr705)(#9145), Acetyl-Stat3 (Lys685)(#2523), JAK1(#3344), JAK2(#3230), PKC(#2056), p53(#2524) from Cell Signaling (Danvers, MA, USA); anti-HA(#H3663), anti-FLAG(#F1804) and anti-β-Actin(A3854) from Sigma-Aldrich (St Louis, MI, USA); Myc (#SC-40) from Santa Cruz (Santa Cruz, CA, USA).

Standard Western blot protocol was adopted. Images were acquired with Tanon-5200 and the density of bands was determined with Image J. The methods for immunoprecipitation was previously described [31].

### GST pull-down assay and in vitro deacetylation assay

pCDNA3.1-GST-*Hdac7*(aa445–938), pCDNA3.1-GST-*Hdac7*(aa445–938)-AWA and pCMV6-*Stat3*-Flag were transfected into 293 T cells separately. Cells were lysed with RIPA (Radio immunoprecipitation assay) buffer 48 h after transfection. GST-Hdac7(aa445–938) and GST-Hdac7(aa445–938)-AWA fusion proteins were purified using Glutathione Sepharose 4B (#17–0756-01 GE Healthcare), and Flag-tagged Stat3 was purified with ANTI-Flag M2 Affinity Gel (#A2220, Sigma) and eluted with 3X FLAG peptide (#F4799, Sigma). For in vitro pull-down assays, purified proteins were incubated together in Nonidet P-40 lysis buffer [31] for 1.5 h, followed by washing with PBS for three times, and finally analyzed by Western blot.

In vitro deacetylation assay was performed as described previously [35] with some modification. In brief, 0.5 μg purified Stat3 protein from 293 T cells was incubated with 0.5 μg purified GST-Hdac7(aa445–938) or GST-Hdac7(aa445–938)-AWA fusion proteins in deacetylation buffer (15 mM Tris–HCl, pH 8.0, 10 mM NaCl, 0.25 mM EDTA, 10 mM 2-mercaptoethanol, 10% (*v*/v) glycerol) for one hour at 37 °C. The reaction products were subjected to Western blot with anti-acetylated lysine antibody.

### Human lung cancer samples and prognosis analysis of lung cancer patients

Clinical lung cancer samples obtained from Huashan Hospital, Fudan University, Shanghai, China were used for immunoblot analysis.

For prognosis analysis of lung cancer patients, gene expression representative as FPKM (fragments Per Kilobase of transcript per Million mapped reads) derived from RNA-seq were downloaded from the TCGA project (https://portal.gdc.cancer.gov/). Kaplan-Meier survival analysis and Cox’s proportional hazards regression were conducted by survival package in R (version: 3.2.3). Prognosis effect from *HDAC7* was estimated by Peto & Peto modification of the Gehan-Wilcoxon test and conducted by survdiff function from survival package (R). Hazard ratio (HR) and corresponding 95%CI were estimated with Cox’s proportional hazards regression.

### Statistical analysis

Statistical analysis was conducted using an unpaired t test by GraphPad Prism (Graphpad, La Jolla, CA, USA). A *p* value < 0.05 was considered significant.

## Results

### *HDAC7* mutant inhibits lung tumorigenesis in mice

**Fig. 1:**
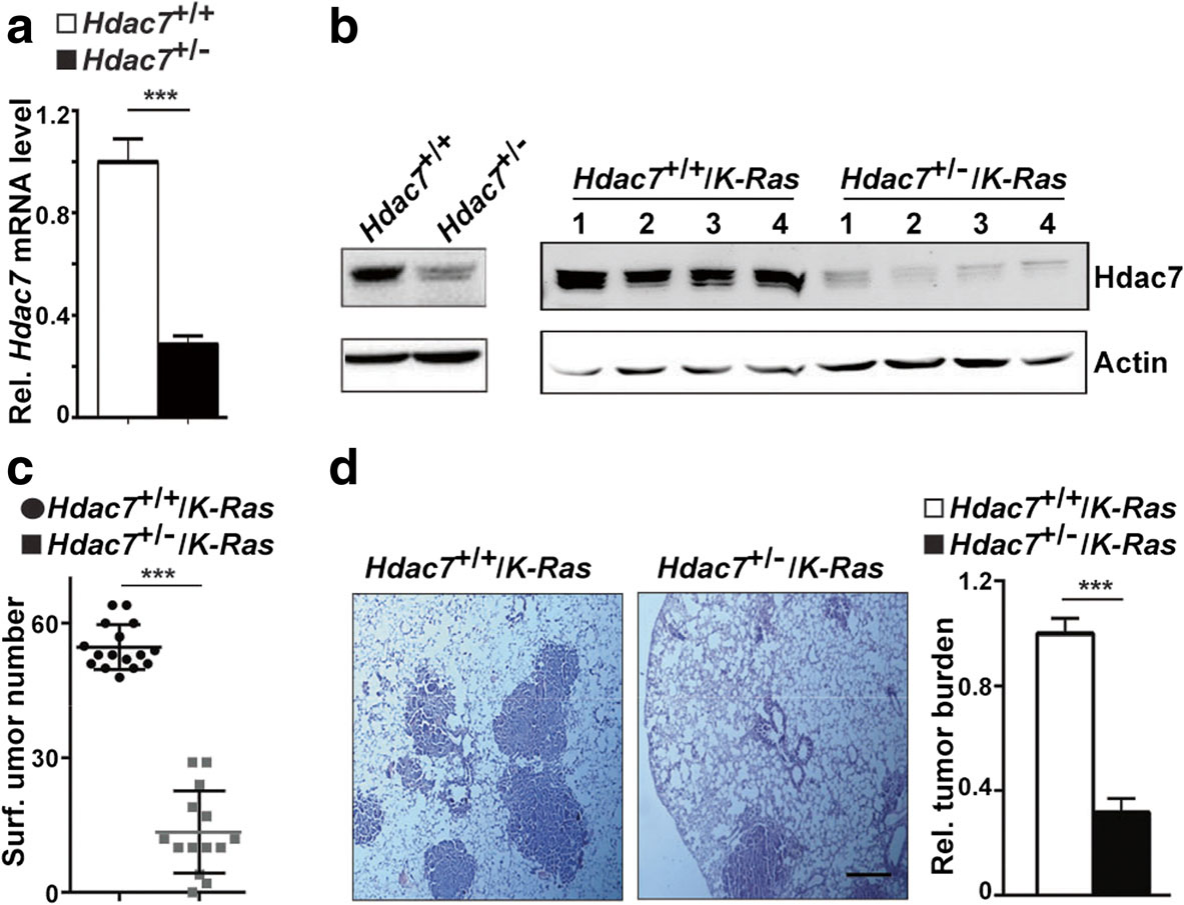
*Hdac7* mutant inhibits lung tumorigenesis in mice. **a**. Statistical analysis of real-time qRT-PCR results for *Hdac7* mRNA lever in normal lungs from wild type and *Hdac7* heterozygous mutant mice. *n* = 3 for each genotypes. **b**. Western blot analyses of the Hdac7 protein level in normal lung tissues of wild type and *Hdac7* ^+/−^ mice (left panel) and lung tumors from *Hdac7* ^+/−^/*K-Ras* and control mice (right panel). Numbers above the blot in right panel represent the individual tumor from different mice. **c**. Statistical analysis of tumor number on mouse lung surface of *Hdac7* ^+/−^/*K-Ras* and control mice. Each dot/square represents an individual mouse. **d**. Histological (left panel) and quantitative (right panel) analyses of tumor burden. Left panel, histological images of H&E stained sections of lungs with tumors from *Hdac7* ^+/−^/*K-Ras* and control mice, *n* = 6 for each genotypes. Bar 0.2 mm. Blots and images shown are representatives of three experiments. Values in (**a** and **d**) represent the means ± SD. of three separate experiments. ***, *p* < 0.001

To investigate the role of *Hdac7* in lung cancer, we first verified *Hdac7* expression in wt and *Hdac7* ^+/−^ mouse lung tissues. The results showed that both *Hdac7* mRNA and protein levels were reduced by more than 50% in *Hdac7* ^+/−^ mice compared to wt mice (Fig. 1a and b left panel). Then, *Hdac7* ^+/−^ mice were crossed with *K-Ras* transgenic mice, the most frequently used mouse model for lung cancer [29]. The resulting *Hdac7* ^+/−^/*K-Ras* mice and control *K-Ras* mice were administrated with Ad-*Cre* to induce lung tumors (see Methods). The results showed that total tumor numbers on mouse lung surface (Fig. 1c) and tumor burden (Fig. 1d) were markedly reduced in *Hdac7* ^*+/−*^ */K-Ras* mice compared with control mice. Immunoblotting also demonstrated again that *Hdac7* expression in *Hdac7* ^+/−^ mouse lung tumors was dramatically decreased (Fig. 1b right panel). These results suggest that *Hdac7* mutant notably inhibits mouse lung tumorigenesis.

### Decreased cell proliferation and increased apoptosis in lung tumors of *Hdac7*^*+/−*^*/ K-Ras* mice

**Fig. 2:**
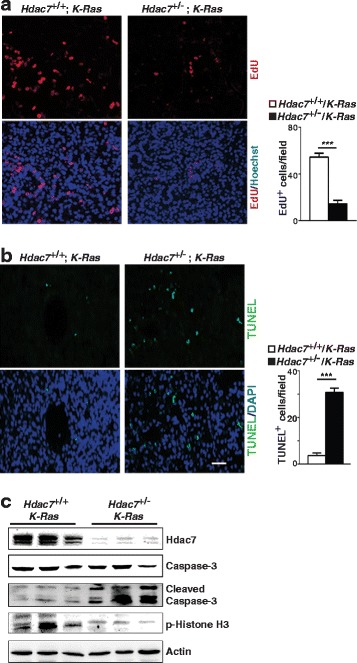
*Hdac7* mutant suppresses proliferation and enhances apoptosis of lung tumor cells in mice. **a**. Left panels: Representative florescent images of mouse lung sections stained for EdU-positive cells (red) in lung tumors. Right panel: Statistics analysis of EdU-positive cells per field. **b**. Left panels: Representative florescent images of mouse lung sections for TUNEL staining of apoptotic cells (green) in lung tumors. Right panel: representative statistics analysis of TUNEL positive cells per field. The genotypes of the mice were indicated above the images. Hoechst and DAPI was used for staining for nucleus, respectively. Bar 50μm. The EdU-positive or TUNEL-positive cells in tumor area of six randomly chosen fields/lung were counted on digital fluorescence images and more than five animals for each genotype were included. Values represent the means ± SD. ***, *p* < 0.001. **c**. Western blotting for caspase-3 cleavage and phosphorylation of Histone H3 in lung tumors from *Hdac7* ^+/−^/*K-Ras* and & control mice

Cell proliferation and apoptosis play critical roles in cancer development. However, previously reported results about the role of *HDAC7* in regulating cell proliferation of normal human umbilical vein endothelial cells (HUVEC) and human cancer cell lines are controversial [36–38] and the functions of *HDAC7* in apoptosis of different cells also appear varied [13, 39, 40]. To understand the cellular mechanism(s) by which *Hdac7* mutant suppresses lung tumorigenesis, we evaluated cell proliferation and apoptosis in lung tumors of *Hdac7* ^*+/−/*^ *K-Ras* and control mice. Our in vivo EdU-pulse labeling experiments showed that cell proliferation was dramatically decreased in *Hdac7* ^*+/−*^ */K-Ras* tumors (Fig. 2a). Our TUNEL assay also revealed that apoptosis in *Hdac7* ^*+/−*^ */K-Ras* tumors was substantially increased (Fig. 2b). Consistent with the results of TUNEL and EdU labeling assay, more caspase-3 cleavage and less p-Histone H3 was observed in *Hdac7* ^+/−^/*K-Ras* tumor cells respectively (Fig. 2c). Taken together, these data strongly suggest that *Hdac7* mutant can inhibit mouse lung cancer via attenuating cell proliferation and promoting apoptosis.

### *HDAC7* silencing suppresses growth of human lung cancer cells

**Fig. 3:**
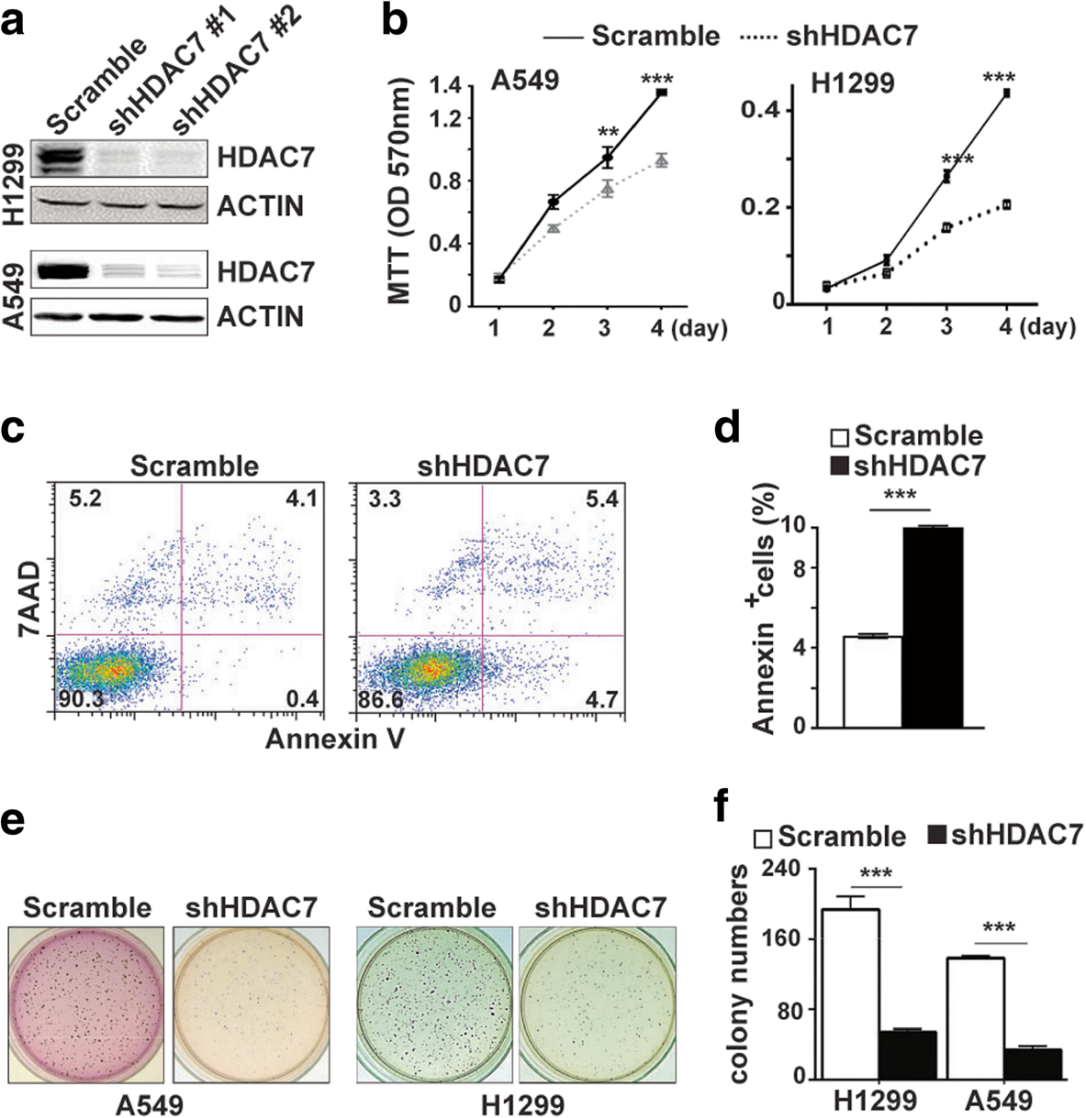
*HDAC7* knockdown suppresses growth of human lung cancer cells. **a**, Representative Western blot of HDAC7 protein levels of human lung cancer cells, H1299 and A549, expressing shHDAC7 and scramble shRNA. **b**. The growth curves of human lung cancer cells, A549 and H1299, infected with lenti-shHDAC7 and control viruses. Cell growth was evaluated by MTT assay. **c-d**. *HDAC7* knockdown induces apoptosis of human lung cancer cells. Representative flow cytometry of H1299/shHDAC7 and the control cells stained with Annexin V and 7AAD (**c**) and the statistical analyses (**d**) of FACS in c. Annexin V positive cells were counted as apoptotic cells. **e-f**. *HDAC7* knockdown inhibits tumorigenecity of human lung cancer cells. Representative photographs of the soft agar colony formation assay for lung cancer cell lines, H1299 and A549, expressing shHDAC7 and scramble shRNA (**e**), and the statistical analyses (**f**) of the results of colony numbers formed in soft agar in e. Values in (**b**, **d** and **f**) represent the means ± SD. of three separate experiments. **, *p* < 0.01;***, *p* < 0.001

Since in vivo study demonstrates that *HDAC7* functions as an oncogenic factor in mouse lung cancer, we further reasoned that reduction of *HDAC7* expression in human lung cancer cells could inhibit their growth. To verify this possibility and exam the biological impact of *HDAC7* depletion on human lung cancer cells, we silenced *HDAC7* expression by lentivirus-mediated shRNA in two human lung cancer cell lines (H1299 and H522) with wt *K-RAS* and two human lung cancer cell lines (A549 and H2009) with a mutant *K-RAS* to mimic the conditions of our mouse study (Fig. 3a and Additional file 1: Figure S1B). The growth of H1299 and A549 cells expressing shHDAC7 was significantly inhibited (Fig. 3b). Annexin V assay showed that *HDAC7* knockdown resulted in significant enhanced apoptosis of human lung cancer cells (Fig. 3c and d). Moreover, soft agar colony formation assay revealed that shHDAC7 markedly suppressed colony formation of H1299 and A549 cells in soft-agar (Fig. 3e and f). These results indicate that suppressing *HDAC7* expression in human lung cancer cells may restrain human lung cancer development.

### Loss of *HDAC7* enhances STAT3 phosphorylation

Knockdown *HDAC7* in multiple human cancer cell lines resulted in suppression of cell growth by down regulating *c-MYC* expression and up regulating *p21* and *p27* expression [38]. *HDAC7* has also been shown to protect neurons from apoptosis by inhibiting *c-JUN* expression [39]. To investigate whether *Hdac7* may regulate the proliferation and apoptosis of lung cancer cells by similar mechanisms, the expression of *c-Myc*, *p21* and *c-Jun* in mouse lung tumors of *Hdac7* ^*+/−*^ */K-Ras* mice were evaluated by immunoblotting. The results showed that the protein levels of c-Myc, c-Jun and p21 were not considerably changed in *Hdac7* ^*+/−*^ */K-Ras* lung tumors compared with the control *K-Ras* lung tumors (Additional file 1: Figure S1A).

**Fig. 4:**
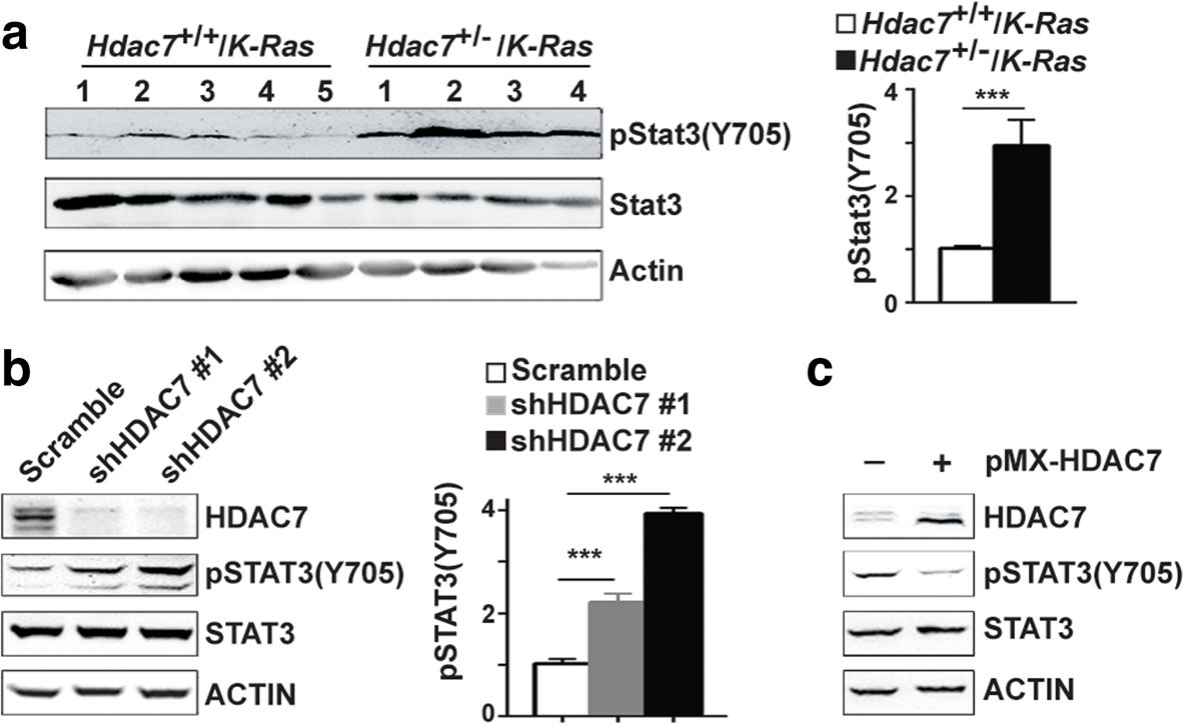
HDAC7 regulates STAT3 phosphorylation. **a**. Representative Western blot (left panel) and statistics analyses of the phosphorylation of Stat3 proteins in individual lung tumors isolated from *Hdac7* ^+/−^/*K-Ras* and control mice. **b**, Representative Western blot (left panel) and quantitative (right panel) analyses of Stat3 phosphorylation level in H1299 cells expressing shHDAC7 and scramble shRNA. **c**, HDAC7 overexpression significantly abrogates STAT3 phosphorylation. Representative Western blot analyses of STAT3 phosphorylation level in H1299 cells transfected with pMX-*Hdac7* and pMX control plasmids. The values in statistics analyses in (**a** and **b)** represent the means ± SD. of three separate experiments. ***, *p*<0.001

Since *HDAC7* silencing was reported to result in activation (phosphorylation of Tyr705) of STAT3 in HUVEC [41], we ask whether *Hdac7* deficiency could also enhance STAT3 phosphorylation in mouse and human lung tumor cells. Indeed, our studies showed that Stat3 tyrosine phosphorylation, but not Stat3 protein level, was markedly increased in lung tumors from *Hdac7* ^*+/−*^ */K-Ras* mice compared to that in lung tumors from control mice (Fig. 4a). Similar results were also observed in multiple human lung cancer cell lines such as H1299, H2009, H522 and A549, expressing *shHDAC7* (Fig. 4b, Additional file 1: Figure S1B). Consistent with the above results, STAT3 phosphorylation was significantly decreased without obvious change of Stat3 protein level when exogenous *Hdac7* was expressed in H1299 (Fig. 4c). These results demonstrate that *Hdac7* negatively regulates Stat3 phosphorylation (activity) without affecting its protein level in mouse and human lung cancer cells.

### *dnStat3* reverses tumor inhibition triggered by *Hdac7* mutant in mice

**Fig. 5:**
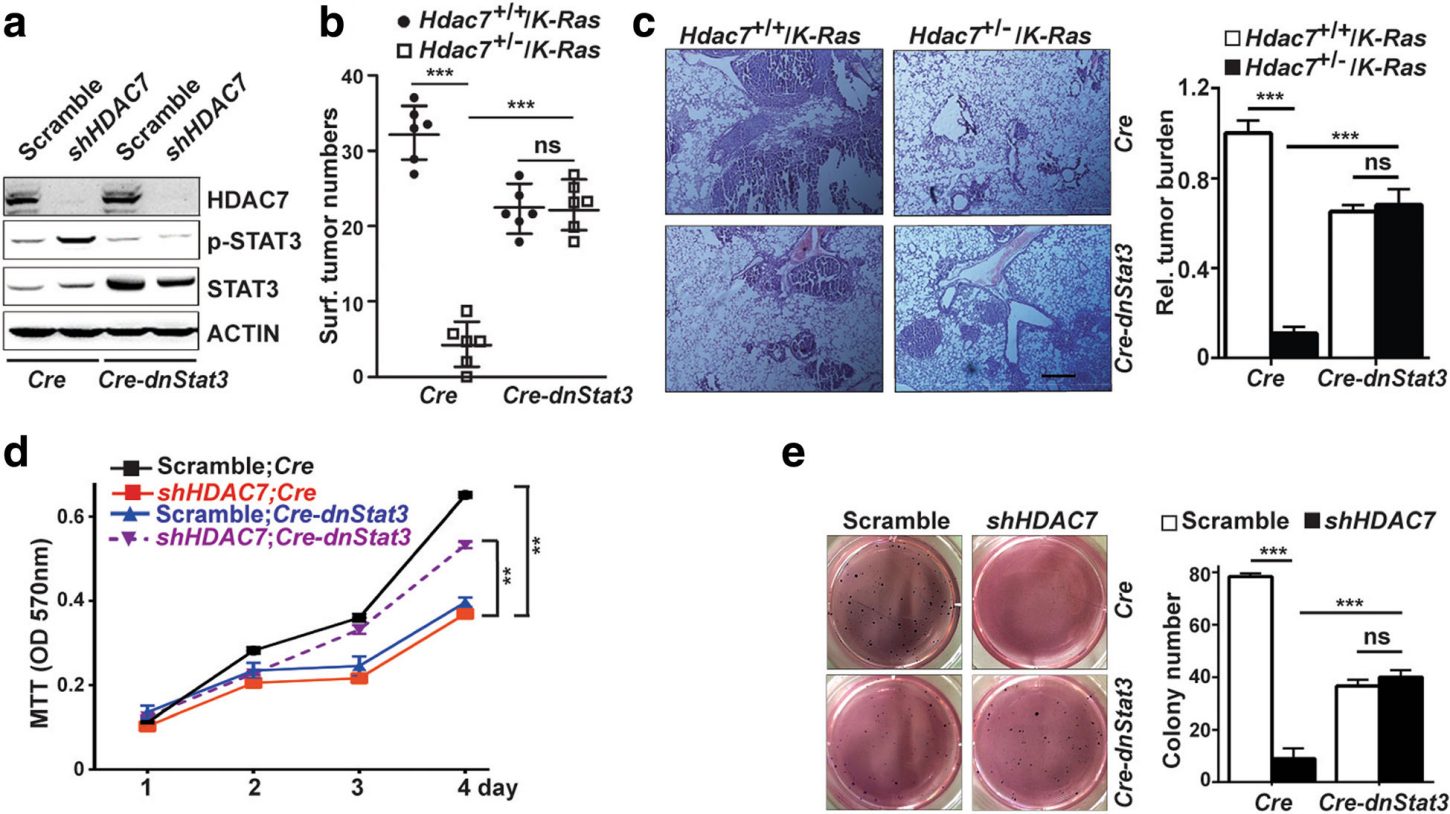
*dnStat3* reverses *Hdac7* mutant –mediated tumor inhibition in mice and partially rescues *HDAC7* depletion-mediated growth suppression of human lung cancer cells. **a** Representative Western blot analysis of the STAT3 protein level and phosphorylation of endogenous STAT3 proteins of shHDAC7 or scramble shRNA-expressing H1299 cells infected with lenti-*Cre* or lenti-*Cre*-2A-*dnStat3* viruses, respectively. **b** Statistical analyses of tumor numbers on lung surfaces of *Hdac7* ^+/−^/*K-Ras* and control mice infected with Lenti-*Cre* or Lenti-*Cre*-2A-*dnStat3* viruses, respectively. Each dot/square represents an individual mouse. **c** Representative histologic images of H&E stained lung sections (left panels) and statistical analyses (right panel) of tumor burden (right panel) of the same mice in (**b**). **d** The growth curves of the same lung cancer cells in (**a**). MTT assay showed that *HDAC7* knockdown-mediated cell growth suppression was partially restored by expressing *dnStat3*. **e**. Representative photographs (left panel) and statistical analysis (right panel) of the soft agar colony formation assay for the same lung cancer cells in (**a**). Values in (**b-e**) represent the means ± SD of three experiments. **, *p* < 0.01; ***, *p* < 0.001; ns, not significant

STAT3 constitutive activation is usually associated with tumor promoting [16]. However, high pY-STAT3 was reported to be highly correlated with a better prognosis for some human cancers [19–21] and *Stat3* can function as a tumor suppressor in *Apc* mice [22] and in *K-Ras* mice [23]. Here we also show that elevated Stat3 phosphorylation is correlated with suppression of lung tumor in *Hdac7* ^*+/−*^ */K-Ras* (Fig. 1c and d). Based on the above information, we hypothesized that *Hdac7* mutant inhibits lung tumorigenesis in *Hdac7* ^*+/−*^ */K-Ras* mice via activating STAT3. To test our hypothesis, we investigated if expression of *Stat3* ^*Y705F*^, a *dnStat3*, could rescue lung tumor-suppression role of *Hdac7* mutant in mice. Two lentiviruses, Lenti-*Cre* expressing *Cre* only and Lenti-*Cre-*2A-*dnStat3* co-expressing *Cre* and *dnStat3,* were constructed. We first verified that expression of *dnStat3* completely abolished enhanced phosphorylation of Stat3 in *Hdac7*-depleted H1299 cells infected by Lenti-*Cre-*2A-*dnStat3* (Fig. 5a, compare lane 4 with lane 2). Then, by tracheal instillation*, Hdac7* ^*+/−*^ */K-Ras* mice and control *K-Ras* mice were infected separately with Lenti-C*re* or Lenti-*Cre-*2A-*dnStat3,* respectively. We found that in the Lenti-*Cre* infected group, *Hdac7* mutant dramatically inhibited lung cancer development, however, in Lenti-*Cre-*2A-*dnStat3* infected group, neither tumor number on lung surface (Fig. 5b) nor tumor burden (Fig. 5c) were significantly different between *Hdac7* ^*+/−*^ */K-Ras* mice and control *K-Ras* mice. These results demonstrate that lung tumorigenesis is significantly enhanced in *Hdac7* ^*+/−*^ */K-Ras* mice after enforced expression of *dnStat3,* that is, that expression of *dnStat3* rescues *Hdac7-*mutant-mediated suppression phenotype of lung tumor in mice. Therefore, we conclude that *Hdac7* mutant suppresses lung tumorigenesis in mice by activating Stat3 proteins.

### *dnStat3* rescues growth suppression triggered by *HDAC7* depletion in human lung cancer cell lines

To verify if *STAT3* also plays a role in human tumor cell growth regulated by *HDAC7*, we tested whether expression of *dnStat3* was able to rescue the growth suppression mediated by *HDAC7* depletion in human lung cancer cell lines. Cell proliferation assay showed that *Hdac7*-depleted H1299 cells grew significantly slower than scrambled H1299 cells when they both were infected with control lentivirus; however, this growth inhibition was recovered or partially recovered (Fig. 5d) when *Hdac7*-depleted H1299 cells were infected lentivirus expressing *dnStat3.* We also found that *dnStat3* expression also partially restored the capacity of colony formation of H1299 cells in soft agar that was suppressed by *HDAC7* depletion (Fig. 5e). These results suggest that *HDAC7* may also regulate the growth of human lung cancer cells by inhibiting STAT3 activity.

### HDAC7 interacts with STAT3 and regulates STAT3 deacetylation

STAT3 phosphorylation is regulated by JAK, Src, PKC kinases [16]. It has been reported that silencing *HDAC7* in glioblastoma (GMB) cell U87 enhanced STAT3 phosphorylation by upregulating JAK1 expression [42]. However, our immunoblotting analysis showed that the expression of JAK1, JAK2 and PKC was not significantly altered when *HDAC7* expression was depleted in human lung cancer cell line H1299 (Additional file 1: Figure S1C).

**Fig. 6:**
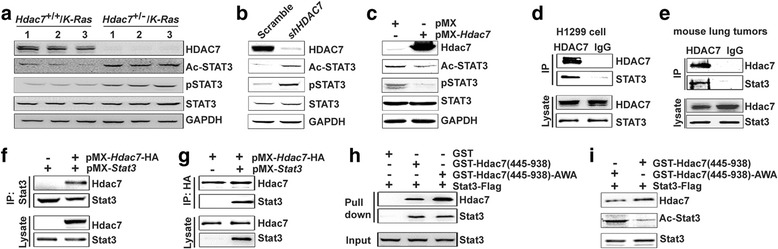
HDAC7 interacts and deacetylates STAT3. **a**-**c**, HDAC7 regulates STAT3 acetylation. Western blot analysis of acetylation and phosphorylation of STAT3 in the individual lung tumors from *Hdac7* ^+/−^/*K-Ras* and control mice (**a**), H1299 human cancer cells expressing shHDAC7 (**b**) and an exogenous mouse *Hdac7* cDNA (**c**) with antibodies specific for Stat3, or acetyl-STAT3(K685) or phospho-Stat3(p-Y705). **d**-**g** In vivo interaction of Hdac7 and Stat3 proteins. Immunoprecipitation -Western blots analyses of the endougenous HDAC7 and STAT3 in H1299 human cancer cells (**d**) and lung tumors from control *K-Ras* mice (**e**), and the exogenous mouse Hdac7 and Stat3 proteins expressed in 293 T cells (**f** and **g**) with the antibodies as indicated. **h** GST pull down-Western blot assay of affinity-purified GST-Hdac7 fusion protein and Flag-tagged Stat3 protein. Anti-Hdac7 and -Stat3 were used for blot to detect GST-Hdac7 fusion proteins and Stat3 protein, respectively. **i** In vitro deacetylase assay with affinity purified GST-HDAC7(aa 445–938), GST-HDAC7(aa 445–938)-AWA and Flag-tagged Stat3 proteins. Acetyl-STAT3 was detected with pan anti-acetyl-Lysine antibodies. Images are representatives of three experiments

STAT3 acetylation can be modulated by histone acetyl transferases/HDACs, e.g. p300/HDAC3, affect its dimerization, DNA binding and transactivation [14]. Also, the STAT3 mutated at key acetylation sites impairs tyrosine-phosphorylation of STAT3 [15]. HDAC7 was reported to repress STAT3 transcriptional activity by forming a complex with histone acetyltransferase Tip60, but there were no provided detailed mechanism(s) [43]. Therefore, we hypothesized that HDAC7 negatively regulates STAT3 activity by deacetylating STAT3. To test our hypothesis, we first evaluated STAT3 acetylation status in both lung tumors from *Hdac7* ^*+/−*^ */K-Ras* mice and *HDAC7*-depleted human lung cancer cell lines. The result revealed that Stat3 acetylation was significantly elevated in *Hdac7* ^*+/−*^ mouse lung tumors (Fig. 6a) and *HDAC7*-depleted H1299 (Fig. 6b) and A549 cells (Additional file 1: Figure S1E). We also found that the STAT3 acetylation was decreased when exogenous *Hdac7* was expressed in human cancer cells, H1299 (Fig. 6c) and A549 (Additional file 1: Figure S1F). Next, we examined if Hdac7 and Stat3 proteins interacted each other. Our study showed that not only exogenous Stat3 and Hdac7 proteins expressed in 293T cells (Fig. 6f and g) were co-immunoprecipitated reciprocally, but also endogenous STAT3 and HDAC7 proteins in human lung cancer cells and mouse lung tumors from control *K-Ras* mice (Fig. 6d and e). This suggests that Hdac7 may directly interact with Stat3 and catalyzes its deacetylation. To further verify this possibility, we performed pull-down assay and in vitro deacetylase assay using affinity purified Flag-tagged Stat3 protein and GST-Hdac7 fusion protein. Since we failed to purify full length GST-HDAC7 fusion proteins and ~500 aa C-terminal domain of HDAC7 has deacetylase activity [44], C-terminal domain (aa 445–938) of mouse Hdac7 protein (NP_062518) was fused to GST and used for the following two experiments. The pull-down assay disclosed that both GST-Hdac7(aa445–938) and GST-Hdac7(aa445–938)-AWA (a mutant *Hdac7* lacking of deacetylase activity [45]), but not GST, bound to Stat3 (Fig. 6h). Our in vitro deacetylase assay also showed that purified GST-Hdac7(aa445–938) but not GST-Hdac7(aa445–938)-AWA, dramatically reduced the acetylation of purified Flag-Stat3 (Fig. 6i). Taken together, these results strongly suggest that the Hdac7 protein directly interacts with and deacetylates Stat3, which in turn regulates Stat3 phosphorylation.

### Upregulated HDAC7 expression in human lung cancer correlates with poor prognosis

**Fig. 7:**
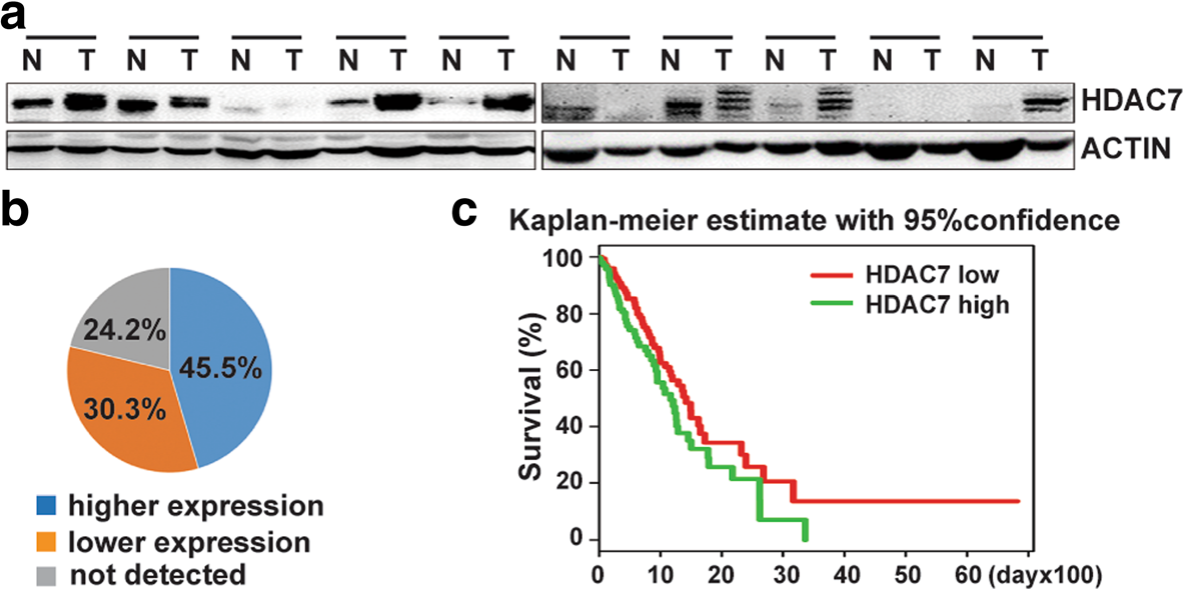
High expression of *HDAC7* in lung cancers correlates with poor patient survival. **a** and **b**. The assess of HDAC7 protein expression in human lung cancer tissues. Representative Western blot analyses of HDAC7 protein level in human lung tumor and adjacent tissues (**a**) and the graph illustration of HDAC7 expression level of 34 human lung cancer tissues (**b**). N: normal adjacent lung tissue, T: lung tumor tissue. **c**, Kaplan-Meier survival analysis of 484 lung cancer patients with low and high *HDAC7* mRNA level in lung cancer tissues (original data from TCGA)

To further investigate the effects of *HDAC7* on human lung cancer, we examined HDAC7 protein level in 33 human lung tumors. HDAC7 expression was upregulated in 15 samples (~45.5%) and down-regulated in 10 samples compared to that in tumor-adjacent tissues, while no HDAC7 protein could be detected in both lung tumors and tumor-adjacent tissue from 8 patients (~24.2%) (Fig. 7a and b). To further gain insight into the role of *HDAC7* in human lung cancer, gene expression data of 484 lung cancer patients were collected from TCGA (the Cancer Genome Atlas) for integrated analysis to assess correlation between *HDAC7* expression and human lung cancer prognosis. As shown in Fig. 7c, Kaplan-Meier survival analysis showed low HDAC7 (< median) is a significant good prognosis factor (*p* < 7.8 × 10^−5^, *X* ^*2*^ = 15.6, df = 1, Gehan-Wilcoxon test) compared with HDAC7 high expression (> median) in overall patients with HR = 1.46 (95%CI: 1.00–2.13, *p* = 0.048). Collectively, these results suggest that up-regulation of *HDAC7* expression function as an oncogenic factor for human lung cancer development.

## Discussion

Lung cancer is still the leading cause of cancer deaths worldwide [46] due to lack of fully understanding of the molecular mechanisms of lung cancer development. Here we demonstrate that both lung tumor number and burden are dramatically reduced in *Hdac7* ^+/−^/*K-Ras* mice compared with those in control *K-Ras* mice. We show that *HDAC7* silencing inhibits cell proliferation and anchorage-independent growth of human cancer cell lines. We have also observed higher HDAC7 protein level in ~ 44% human lung tumor samples and found that high *HDAC7* mRNA level in human lung cancer is correlated to poor prognosis. All these results from studies of mouse genetics, human lung cancer cell lines and clinic lung cancer patients strongly suggest that *HDAC7* play an oncogenic role in human lung cancer, but our conclusion is contradictory to a previous report by Osada, et al., claiming that high *HDAC7* mRNA level in human lung tumors was correlated to good progonosis [4]. One possible reason for the discrepancy may come from the different samples size in these two studies. Only 72 human lung cancer samples were analyzed by Osada, et al., while data from 484 lung cancer samples from TCGA were evaluated by this study.

*HDAC7* enhances the proliferation of HUVEC and cancer cells such as HeLa, HCT116 and MCF-7 probably by stimulating c-*Myc* and inhibiting *p21* and *p27* expression [37, 38]. *HDAC7* can also protect mouse thymocytes and cerebellar granule neurons from apoptosis via repressing *Nur77* and *c-Jun* expression, respectively [39, 47]. In contrast, *HDAC7* has recently been reported to promote apoptosis of human pro-B-ALL and Burkitt lymphoma by down regulating *c-Myc* expression [13]. Our study shows that reduction of *Hdac7* expression in mice results in a decreased proliferation and increased apoptosis of mouse lung cancer cells. However, we only observed enhanced phosphorylation (activation) of Stat3 proteins but no obvious alteration at protein levels for Stat3, c-Myc, c-Jun, p21 and p53 (Additional file 1: Figure S1A). All these results suggest that *HDAC7* may regulate the proliferation and apoptosis of various cells through different molecular mechanisms.

During the period of preparing this manuscript, Peixoto, et al. reported that *HDAC7* high expression in glioblastoma (GBM) is associated with poor prognosis. They have also demonstrated that *HDAC7* silencing suppressed the tumor growth of GBM cell U87 in vivo mainly by inhibiting angiogenesis because *HDAC7* depletion had no effect on the proliferation of GBM cell U87 in vitro [42]. Mechanistically, they also showed that *HDAC7*-depletion inhibited angiogenesis by activating the expression of JAK1 and AKAP12, both of which can synergistically sustain the activity of STAT3 by inducing its phosphorylation (JAK1 tyrosine kinase) and protein expression (AKAP12) [42]. Here we show that mouse *Hdac7* mutation suppresses lung tumor development in vivo and *HDAC7* silencing in human lung cancer cell lines inhibits their proliferation in vitro. Mechanistically, we have demonstrated *Hdac7* can directly interact with Stat3 and deacetylate Stat3 proteins, and decreasing *Hdac7* expression by mutation in mice or shRNA in human lung cancer cells results in enhanced acetylation and phosphorylation of Stat3 without significant effect on the expression of *JAK1* and *AKAP12* (Additional file 1: Figure S1B). Furthermore, STAT3 acetylation but not tyrosine phosphorylation has been shown to be required to silence expression of many tumor suppressor genes such as *SHP-1, CDKN2A* by recruiting DNMT1 to methylate their promoter in cancer cells [48–50]. Therefore, although *HDAC7*-mediated angiogenesis may also play a role in lung tumor development, we think that reducing *Hdac7* expression is an important and sufficient factor to suppress lung tumorigenesis by inhibiting proliferation and enhancing apoptosis of tumor cells in mice, and probably in humans. This notion is supported by our observation that the expressions of cyclin D and cyclin E were significantly decreased in both lung tumors from *Hdac7* ^*+/−*^ */K-Ras* mice (Additional file 1: Figure S1A) and *HDAC7*-depleted human lung cancer cell line H1299 (Additional file 1: Figure S1D). This notion is also further supported by recent findings that *HDAC7* expression is necessary to maintain breast and ovarian cancer stem cells in human and over-expression of *HDAC7* is sufficient to augment the CSC phenotype [11].

Both positive and negative associations between STAT3 activation and survival of lug cancer patients or lung tumor progression have been reported [23, 26, 27]. Furthermore, Zhou, et al., have recently shown that deletion of *Stat3* in *K-Ras* mice enhanced lung tumor number but reduced lung tumor volume, suggesting that *Stat3* can function as a tumor suppressor and an oncogene at different stages of lung tumor development [28]. Their study also showed that cell proliferation and *Cyclin D* expression were decreased in the tumors from their *Stat3* ^*−/−*^ */K-Ras* mice in which *Stat3* was deleted. These results only provided cellular and molecular mechanisms underlying *Stat3* as an oncogene and failed to illustrate the cellular and molecular mechanisms underlying *Stat3* as a tumor suppressor. Here, our study results show that tumor number, tumor burden (Fig. 1c and d), and tumor size (unpublished data) were all decreased in *Hdac7* ^*+/−*^ */K-Ras* mice. Our study supports the notion that *Stat3* function as a tumor suppressor, but do not back the notion that Stat3 can also function as oncogene in lung tumor development because our results revealed that cell proliferation, Cyclin D and cyclin E expression were all decreased when Stat3 was more activated (Y705 phosphorylation) in the tumors from our *Hdac7* ^*+/−*^ */K-Ras* mice. Nevertheless, these two studies suggest that *Stat3* may function as a tumor suppressor in lung cancer under different genetic scenarios.

STAT3β, lacking a transcriptional activation domain at its C-terminal, is a product of differential splicing form of *STAT3* gene and displays a dominant negative function for the STAT3 [51]. Zhang, et al., have shown that high STAT3β expression correlated with a favorable prognosis in patients with esophageal squamous cell carcinoma (ESCC). Expression of STAT3β substantially increased the Y705-phosphorylation, nuclear translocation, and DNA binding/promoter occupation of STAT3, but the transcriptional activity of STAT3 decreased by STAT3β [52]. Here, we have not only shown that tumorigenesis was inhibited in *Hdac7* ^*+/−*^ */K-Ras* mice and Stat3 was more activated (more phosphorylation of Y705) in *Hdac7* ^*+/−*^ */K-Ras* tumors, but we also demonstrated that suppression of endogenous Stat3 activity in lung tumor cells, by expressing *dnStat3,* reversed *Hdac7* mutant-mediated reduction of tumor number and burden in *Hdac7* ^*+/−*^ */K-Ras* mice. These results rule out the possibility that reduced tumorigenesis in *Hdac7* ^*+/−*^ */K-Ras* mice is due to increase expression of Stat3β because, if that is the case, expressing *dnStat3* should result in further reduction rather than enhanced tumorigenesis in *Hdac7* ^*+/−*^ */K-Ras* mice.

The catalytic activity of class IIa HDACs (including HDAC4, −5, −7 and −9) was initially attributed to the presence of class I HDACs co-purified along with the class IIa HDACs. Later, the catalytic domains of HDAC4 and HDAC7 purified from *E. coli* that lacks histones and endogenous HDACs were demonstrated to have a low but measurable deacetylase activity on acetylated-lysine of histones [53, 54] and a high deacetylase activity on trifluoroacetyl lysine, a class IIa-specific substrates in vitro [53–55]. One of possible reasons proposed to explain the low catalytic activity of class IIa HDACs is that acetylated-lysine of histones is not a biological substrate for class IIa HDACs [54]. Now we demonstrate that loss of *Hdac7* resulted in significantly enhanced Stat3 acetylation in both mouse primary tumors and human tumor cell lines. Our co-immunoprecitaion and pull down assay also show that HDAC7 protein directly interacts with Stat3 and the HDAC7 catalytic domain with AWA mutations failed to deacetylate Stat3 in the in vitro deacetylase assay. All these data suggest that the Stat3 protein may be a biological substrate for class IIa deacetylase HDAC7. However, the possibility that contribution of contamination of class I deacetylases such as HDAC3 cannot be ruled out completely since the HDAC7 catalytic domain used for our in vitro deacetylase assay was purified from mammalian 293T cells. Further experiments using HDAC7 purified from *E.coli* and/or class I-selective HDAC inhibitors will help to solve whether Stat3 is a biological substrate for class IIa deacetylase HDAC7.

## Conclusion

Our studies demonstrate that HDAC7 functions as a tumor promoting factor by deacetylating STAT3, therefore reducing STAT3 activation. This furthers our understanding of lung cancer development and may provide a theoretical base for developing new therapeutic strategies for human lung cancer.

## Additional file

Additional file 1: Figure S1. The effects of *Hdac7* on the expression and post-translational modifications of other signaling molecules. A. Western blot and quantitative analyses of c-Jun, c-Myc, P21, P53, Cyclin D and Cyclin E proteins in lung tumors from the *Hdac7*+/−; *K-Ras* and control mice. B. Western blot and quantitative analyses of JAK1, JAK2, PKC-a and AKAP12 proteins in H1299 human lung cancer cells expressing shHDAC7. C. Western blot analysis of STAT3 phosphorylation in human lung cancer cells, A549, H2009 and H522 expressing shHDAC7. D. Representative immunoblot analysis of Cyclin D and Cyclin E in *HDAC7* silencing H1299 cells. E and F. IP-Western blot analysis of STAT3 acetylation in A549 lung cancer cells expressing shHDAC7 (E) or exogenous *Hdac7* with indicated antibodies (F). Ac-lysine: a pan anti-acetyl-Lysine antibodies. Images are representatives of three experiments. Values in A and B represent the means ± S.E of three experiments. **, *p* < 0.01 ***, *p* < 0.001; ns, not significant. (PDF 880 kb)

## Abbreviations

AKAP12: A-kinase anchor protein 12
CSC: Cancer stem cell
DMSO: Dimethyl sulfoxide
EdU: 5-Ethynyl-2′-deoxyuridine
GST: Glutathione S-transferase
H&E: Hematoxylin and eosin
HDAC7: Histone deacetylases 7
JAK1: Janus kinase 1
JAK2: Janus kinase 2
K-Ras: Kirsten rat sarcoma viral oncogene homolog
MTT: 3-(4,5-dimethyl-2-thiazolyl)-2,5-diphenyl-2-H-tetrazolium bromide
PB: PiggyBac
PFU: Plaque-forming units
PKC: Protein kinase C
RNA-Seq: RNA sequencing
STAT3: Signal transducer and activator of transcription 3
TCGA: The Cancer Genome Atlas
Tip60: Tat interactive protein 60 kDa
TU: Transforming unit
TUNEL: Terminal deoxynucleotidyl transferase dUTP nick end labeling
