# Supplementary material for: Hdac7 promotes lung tumorigenesis by inhibiting Stat3 activation
Source: Mol Cancer. 2017 Nov 10;16:170. doi: 10.1186/s12943-017-0736-2 (PMC5681774; doi:10.1186/s12943-017-0736-2)
Supplement: Additional file 1: Figure S1. — The effects of Hdac7 on the expression and post-translational modifications of other signaling molecules. A. Western blot and quantitative analyses of c-Jun, c-Myc, P21, P53, Cyclin D and Cyclin E proteins in lung tumors from the Hdac7+/−; K-Ras and control mice. B. Western blot and quantitative analyses of JAK1, JAK2, PKC-a and AKAP12 proteins in H1299 human lung cancer cells expressing shHDAC7. C. Western blot analysis of STAT3 phosphorylation in human lung cancer cells, A549, H2009 and H522 expressing shHDAC7. D. Representative immunoblot analysis of Cyclin D and Cyclin E in HDAC7 silencing H1299 cells. E and F. IP-Western blot analysis of STAT3 acetylation in A549 lung cancer cells expressing shHDAC7 (E) or exogenous Hdac7 with indicated antibodies (F). Ac-lysine: a pan anti-acetyl-Lysine antibodies. Images are representatives of three experiments. Values in A and B represent the means ± S.E of three experiments. **, p < 0.01 ***, p < 0.001; ns, not significant. (PDF 880 kb) [file 12943_2017_736_MOESM1_ESM.pdf]

Supplementary Figure S1

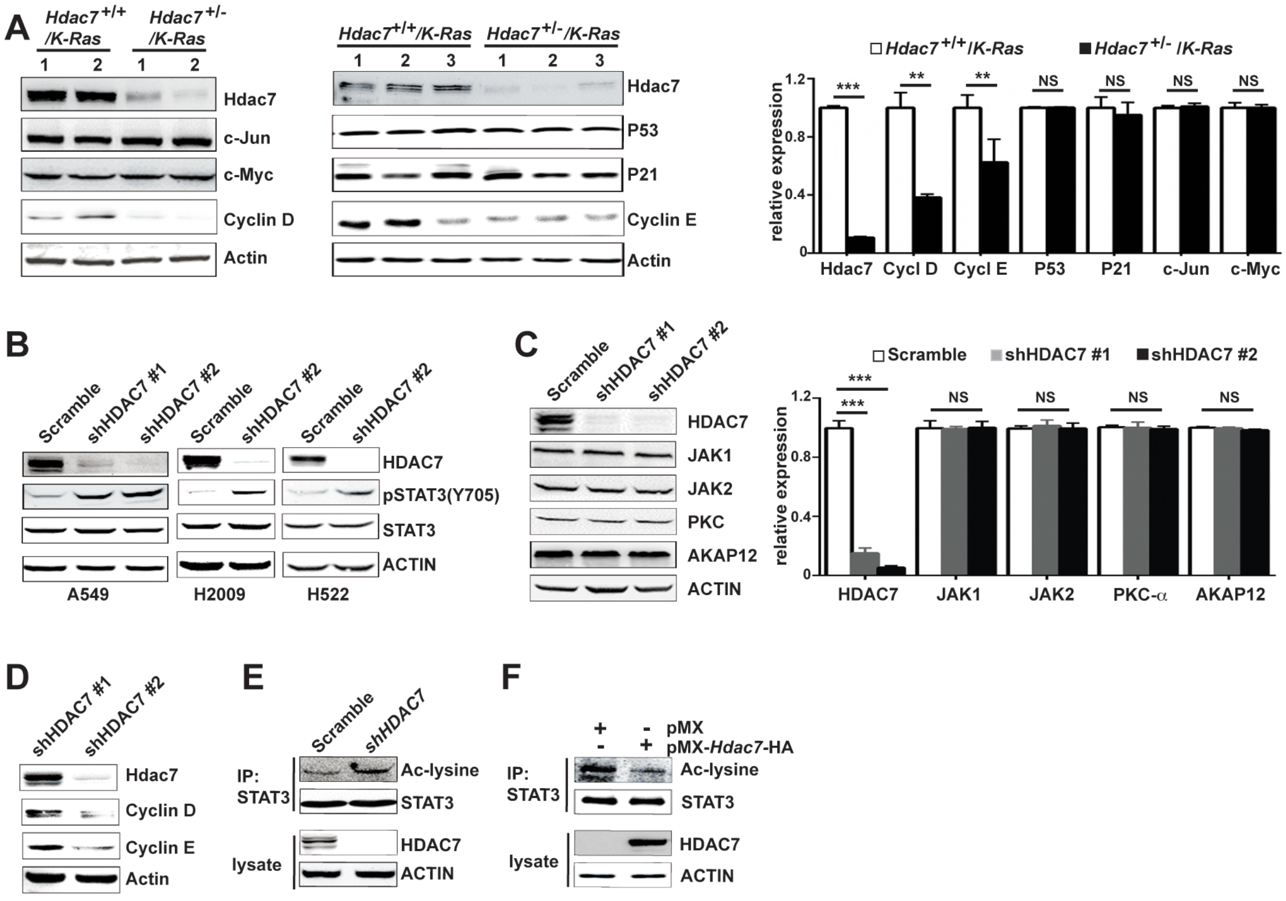

Figure S1. The effects of *Hdac7* on the expression and post-translational modifications of other signaling molecules

A. Western blot and quantitative analyses of c-Jun, c-Myc, P21, P53, Cyclin D and Cyclin E proteins in lung tumors from the *Hdac7*$^{+/-}$; *K-Ras* and control mice. B. Western blot and quantitative analyses of JAK1, JAK2, PKC-a and AKAP12 proteins in H1299 human lung cancer cells expressing shHDAC7. C. Western blot analysis of STAT3 phosphorylation in human lung cancer cells, A549, H2009 and H522 expressing shHDAC7. D. Representative immunoblot analysis of Cyclin D and Cyclin E in *HDAC7* silencing H1299 cells. E and F. IP-Western blot analysis of STAT3 acetylation in A549 lung cancer cells expressing shHDAC7 (E) or exogenous *Hdac7* with indicated antibodies (F). Ac-lysine: a pan anti-acetyl-Lysine antibodies. Images are representatives of three experiments. Values in A and B represent the means ± S.E of three experiments. **, p<0.01 ***, p<0.001; ns, not significant.
